# Uncommon Cause of Internal Mammary Artery Pseudoaneurysm

**DOI:** 10.31486/toj.21.0090

**Published:** 2022

**Authors:** Rohan M. Prasad, Jason Z. Liu, Christopher Garces, Ayushma Duwadi, James Choi, Farah Anwar, Adesuwa Olomu

**Affiliations:** ^1^ Department of Internal Medicine, Michigan State University, Lansing, MI; ^2^ Department of Pulmonology, Michigan State University, Lansing, MI; ^3^ Department of Internal Medicine, Sparrow Hospital, Lansing, MI

**Keywords:** *Angiography*, *fibromuscular dysplasia*, *hematoma–subdural*, *mammary arteries*, *pseudoaneurysm*

## Abstract

**Background:** Internal mammary artery pseudoaneurysms most commonly develop from thoracic penetrating trauma or procedures. However, other important etiologies should not be overlooked.

**Case Report:** A 27-year-old female presented with antiphospholipid antibody syndrome, thrombotic microangiopathy, end-stage renal disease on hemodialysis, and epilepsy. On admission, the patient had pulseless electrical activity and hypertensive emergency. After the patient was successfully resuscitated, she developed status epilepticus. Laboratory workup on admission revealed a subtherapeutic international normalized ratio, elevated C-reactive protein and sedimentation rate, and acute anemia. Imaging showed a right-sided subdural hematoma with a midline shift and likely internal mammary artery pseudoaneurysm. Angiography demonstrated aneurysmal dilation, segmental narrowing, and a string of beads appearance. Because of our patient's demographics, string of beads appearance on diagnostic angiography, history of renal disease, and negative hepatitis serology, fibromuscular dysplasia was considered the etiology of the internal mammary artery pseudoaneurysm. The family opted for 2 burr holes and a subdural drain but declined further diagnostic and therapeutic interventions because of anoxic brain injury and poor prognosis.

**Conclusion:** In this patient, the etiology of the internal mammary artery pseudoaneurysm was attributed to fibromuscular dysplasia. Although this patient's family chose comfort measures, treatment methods are available for internal mammary artery pseudoaneurysms.

## INTRODUCTION

Disruption of arterial wall continuity can lead to blood dissecting into tissues and forming a pseudoaneurysm. A pseudoaneurysm, also known as a false aneurysm, is a perfused sac that communicates with the arterial lumen and can develop in arteries as the result of traumatic, iatrogenic, and inflammatory causes. Pseudoaneurysm of the internal mammary artery is a rare but well-known complication of sternotomy, insertion of central venous catheter or cardiac pacemaker leads, penetrating chest trauma, and percutaneous biopsy. Idiopathic cases and those caused by blunt trauma, vasculitis, atherosclerosis, connective tissue disorders, and adjacent infections are less prevalent.^1-3^ We report a case of internal mammary artery pseudoaneurysm in a patient with fibromuscular dysplasia. This report was prepared following the CARE guidelines.^4^

## CASE REPORT

A homeless 27-year-old Caucasian female presented for unresponsiveness. She had a history of antiphospholipid antibody syndrome on warfarin, thrombotic microangiopathy, deep vein thrombosis, stroke, epilepsy, resistant hypertension, end-stage renal disease on hemodialysis, anemia of chronic disease, and medication noncompliance. Further history and review of systems could not be obtained, as the patient was somnolent and difficult to arouse. Initial vital signs were blood pressure 206/145 mm Hg, heart rate 91/min, and 85% oxygen saturation on room air. In the emergency department, the patient had hypertensive emergency and pulseless electrical activity. Eight hours after a successful resuscitation, she developed status epilepticus.

**Figure 1.**
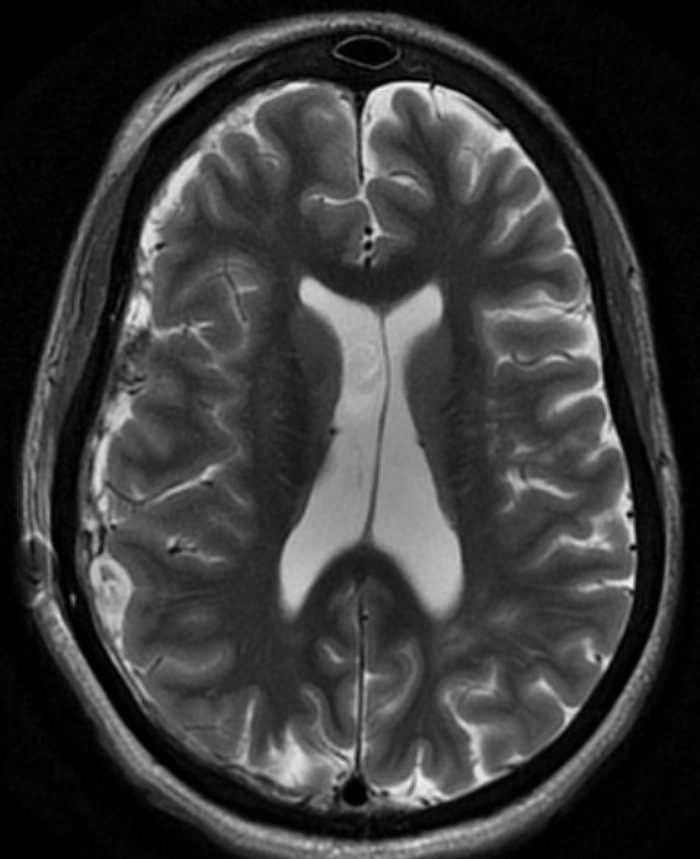
Magnetic resonance imaging of the head on admission showed right-sided subdural hematoma, 5.8-cm midline shift, and cortical laminar necrosis.

**Figure 2.**
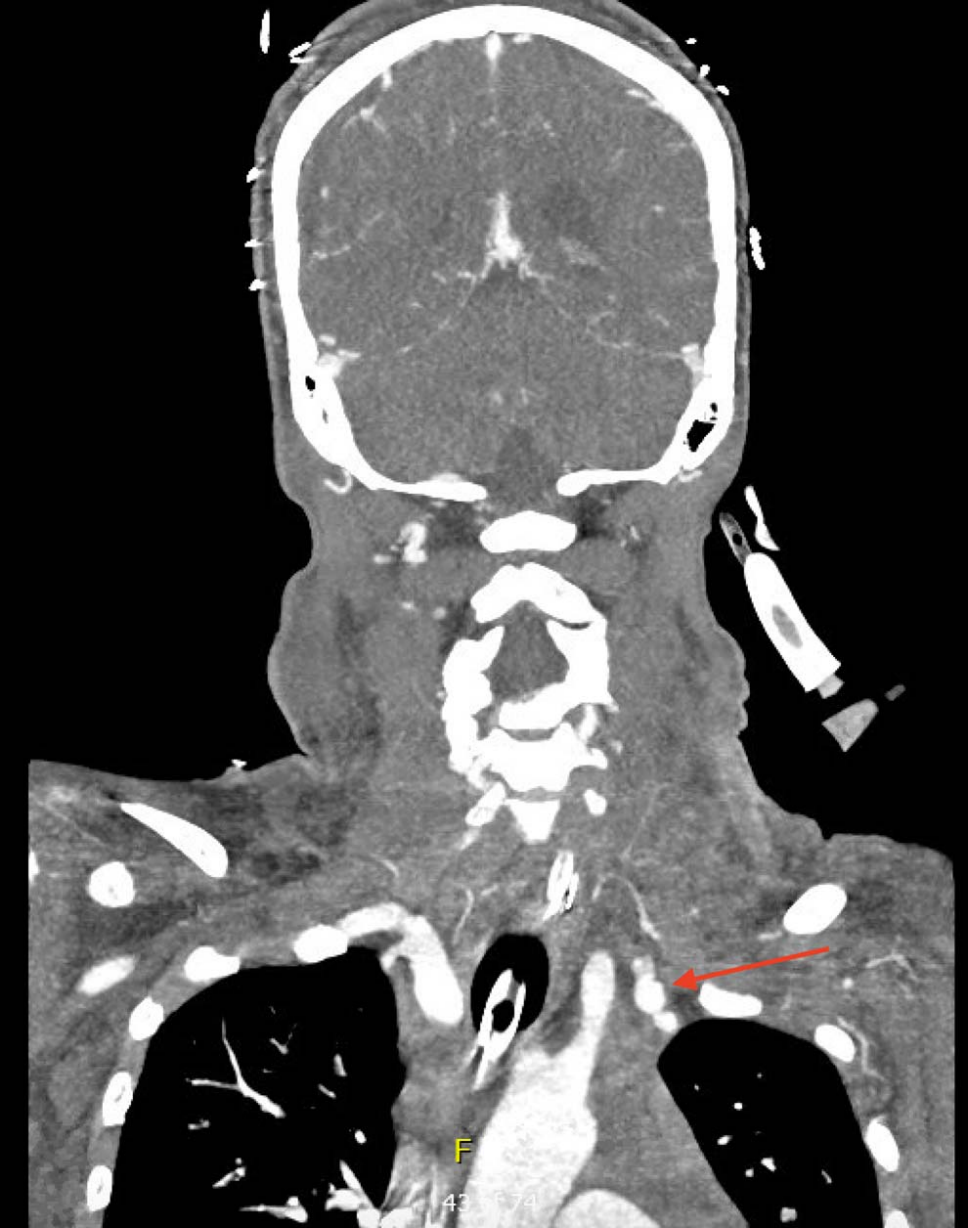
Computed tomography with contrast of the head and neck on day 2 (coronal view) found a dilated left internal mammary artery with possible bleeding (arrow).

**Figure 3.**
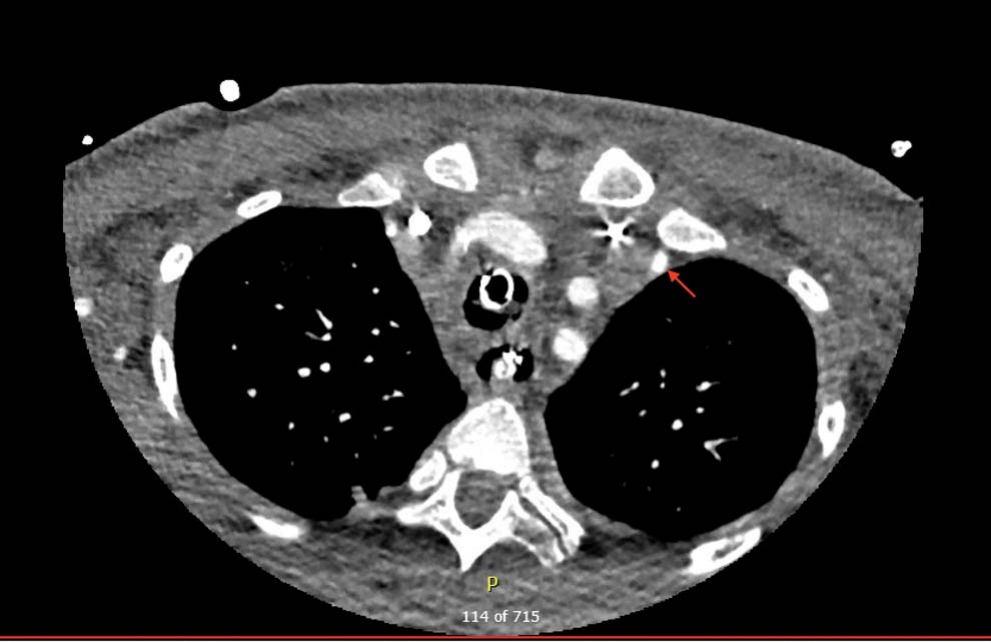
Computed tomography with contrast of the head and neck on day 2 (axial view) demonstrated dilated left internal mammary artery with possible bleeding (arrow).

**Figure 4.**
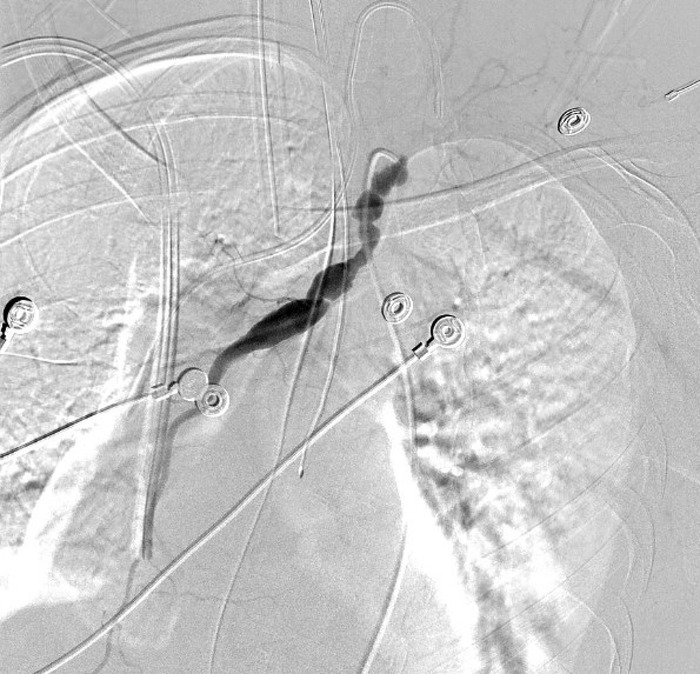
Interventional radiology angiography of the chest on day 2 showed pseudoaneurysmal dilatation, segmental narrowing, and web formation of the artery that resembled a string of beads without bleeding.

Laboratory investigations showed an acute drop in her anemia of chronic disease, subtherapeutic international normalized ratio, and elevated C-reactive protein (CRP) and erythrocyte sedimentation rate (ESR) (Table 1). The patient was started on a nicardipine drip (dose was titrated from 0-15 mg/h to maintain systolic blood pressure of <160 mm Hg for 44 hours), levetiracetam (loaded with 1 g intravenous [IV] and continued on 500 mg IV every 12 hours), and lacosamide (200 mg IV every 12 hours). Computed tomography (CT) without contrast and magnetic resonance imaging of the head revealed a right-sided subdural hematoma with a 1.1-cm midline shift that rapidly progressed to 5.8 cm in 4 hours (Figure 1). The patient received vitamin K (10 mg IV once), 2 burr holes, and a subdural drain. Electroencephalogram showed multiple electrographic seizures with generalized onset, spike waves, and periodic discharges consistent with generalized nonconvulsive status epilepticus (Table 2). CT of the head and neck with contrast on day 2 found an internal mammary artery pseudoaneurysm with possible bleeding (Figures 2 and 3) that was not seen on a chest CT angiography done 3 years prior to presentation. Subsequent diagnostic angiography showed pseudo-aneurysmal dilatation, segmental narrowing, and web formation of the artery that resembled a string of beads without bleeding (Figure 4). Renal ultrasound from 2 years prior to presentation from an out-of-network hospital revealed bilateral echogenic kidneys, consistent with the patient's end-stage renal disease.

**Table 1.** Initial Laboratory Investigations

Test	Patient's Admission Value	Institutional Reference Range
International normalized ratio	1.4	2-3
Erythrocyte sedimentation rate, mm/h	98	0-20
C-reactive protein, mg/dL	7.3	0-1
Hemoglobin, g/dL	5.4	12-15
Iron, μg/dL	37	50-150
Total iron binding capacity, μg/dL	188	270-440
Iron saturation, %	20	20-250
Ferritin, ng/mL	540	7-292

**Table 2.** Diagnostic Examinations and Findings

Test	Day of Hospital Course	Result
Computed tomography of the head without contrast	1	Mixed density right subdural hematoma with chronic and acute elements Left midline shift of 1.1 cm
Magnetic resonance imaging of the head without contrast	1	Right subdural hematoma overlying frontal, parietal, and temporal lobes with 5.8-cm left midline shift Small mixed-signal left epidural hematoma over left parietal lobe
Magnetic resonance angiography/venography of the neck	1	Normal blood flow
Computed tomography of the head without contrast	2	Interval worsening of sulcal effacement throughout the right cerebral hemisphere with concerns for loss of gray-white differentiation Interval decrease in right subdural hematoma and left epidural hematoma after surgery
Electroencephalogram	2	Multiple electrographic seizures with generalized onset, interictal epileptiform discharges with generalized spike waves, and generalized periodic discharges
Computed tomography angiogram of the head and neck	3	New anterior mediastinal soft tissue density with an irregular appearance along left internal mammary artery, suspicious for a pseudoaneurysm with minimal active bleeding Consolidation within the superior segment of the left lower lobe
Diagnostic angiography	4	Aneurysmal dilatation, segmental narrowing, and web formation of the left internal mammary artery

We attributed this patient's internal mammary artery pseudoaneurysm to fibromuscular dysplasia based on her demographics, CT angiography findings, end-stage renal disease, and no recent procedures. However, we could not rule out the possibility of the etiology being secondary to polyarteritis nodosa, as she had resistant hypertension, antiphospholipid syndrome, and elevated levels of CRP and ESR. Additionally, although unlikely, the trauma from the cardiopulmonary resuscitation necessitates consideration as the cause of the internal mammary artery pseudoaneurysm.

Because of the suspicion of anoxic brain injury, the family declined further investigation of vasculitis etiology and treatment of the pseudoaneurysm. They opted for comfort care measures, and the patient died in the intensive care unit on day 6 of hospitalization.

## DISCUSSION

The internal mammary artery arises from the subclavian artery and has collateral arteries to supply the anterior intercostal branches, distal part of the superior epigastric artery, and musculophrenic artery. The internal mammary artery is commonly used in coronary artery bypass graft surgery.^1-3^ Within the upper intercostal space, the internal mammary artery travels close to the pleura and anteriorly to the transversus thoracic muscle, with its final destination being the sixth intercostal space. Through its anatomic course, the internal mammary artery is vulnerable to severe deceleration or penetrating injuries.^3^ Furthermore, the internal mammary artery is located within a vacuous thoracic cavity and is therefore not surrounded by significant amounts of supporting tissue and is in proximity to the dynamic motion of the chest wall. These factors create an ideal environment for the development and possible rupture of an internal mammary artery pseudoaneurysm.^1^ Although nontraumatic internal mammary artery pseudoaneurysms are rare, a possible mechanism of development is the loss of elasticity resulting from structural vascular wall changes, such as those caused by cystic medial necrosis or hyperplasia.^3^

Typical symptoms of internal mammary artery pseudo-aneurysm are isolated progressive chest pain, cough, dys-pnea, hemoptysis, self-limiting hematoma, supraclavicular or intercostal mass, continuous murmur, continuous vibration in the chest, and parasternal painful swelling. More symptomatic and lethal complications include profuse bleeding, hemomediastinum, hemothorax, hemopneumothorax, pneumothorax, and hemorrhagic shock. The dilatation from the pseudoaneurysm can possibly compress the phrenic nerve and cause palsy and dysfunction of the diaphragm.^1,3^

Most internal mammary artery pseudoaneurysms develop from thoracic trauma or procedures. Specifically, the prevalence is 28.8% from sternotomies, 13.6% from central venous catheter insertion, 13.6% from cardiac pacemaker lead insertion, 27.1% from blunt chest trauma, and 11.9% from penetrating chest trauma.^5^ Other less common causes of internal mammary artery pseudoaneurysm include fibromuscular dysplasia,^5^ polyarteritis nodosa,^6^ Kawasaki disease,^7^ systemic lupus erythematosus,^8^ Marfan syndrome,^9^ Ehlers-Danlos syndrome,^10^ type 1 neurofibromatosis,^11^ and chest wall infections.^12^

The diagnosis of internal mammary artery pseudo-aneurysm requires confirmation by chest CT angiography. Consistent chest x-ray findings of internal mammary artery pseudoaneurysm include anterior retrosternal blood effusion producing a defined outline or D-shape with blood pooling in the anterior mediastinum, causing a widened mediastinum appearance. With internal mammary artery pseudoaneurysm, an elevated diaphragm from phrenic nerve palsy can also be appreciated on chest x-ray.^1^ The presence of a string of beads or sausage-like dilatation on CT angiography indicates alternating areas of stenosis and poststenotic aneurysmal dilatation from the internal mammary artery pseudoaneurysm.^13,14^

Once an internal mammary artery pseudoaneurysm is diagnosed, determining the etiology of the disease is important: trauma, postprocedure, fibromuscular dysplasia, or polyarteritis nodosa. Histologic evaluation is required to definitively differentiate between fibromuscular dysplasia or polyarteritis nodosa, but certain presentations may make one more likely. Patients with fibromuscular dysplasia are typically young Caucasian females without systemic symptoms who have negative hepatitis serology and normal levels of acute phase reactants, except in cases of acute infarct.^13^ Histologically, fibromuscular dysplasia is classified as nonatherosclerotic, noninflammatory angiopathy that affects small and medium-sized vessels.^13^ A typical scenario of polyarteritis nodosa is a 50-year-old male with symptoms of weight loss, arthralgias, fatigue, and abdominal pain.^13^ Additionally, signs of hypertension, renal infarction, dissection, stroke, elevated CRP and ESR, and prevalence of medium-sized vessel predilection are commonly seen in patients with polyarteritis nodosa.^13,14^

With our patient's demographics, string of beads appearance on diagnostic CT angiography, history of renal disease, negative hepatitis serology, and absence of recent procedures, fibromuscular dysplasia was the likely etiology. However, polyarteritis nodosa could not be effectively ruled out because of her resistant hypertension, strokes from antiphospholipid antibody syndrome, and persistently elevated levels of CRP and ESR (6.7 mg/dL and >100 mm/h, respectively, from 2 years prior to presentation). Additionally, the patient received cardiopulmonary resuscitation—which can cause blunt chest trauma—for pulseless electrical activity. A connection between cardiopulmonary resuscitation–induced trauma and internal mammary artery pseudoaneurysm has not been reported in the large-scale systematic reviews evaluating the etiology of the pseudo-aneurysm, but they were linked in a case report.^15^ In our case, a link between internal mammary artery pseudo-aneurysm and cardiopulmonary resuscitation is unlikely as the patient's unresponsiveness at presentation was attributed to the internal mammary artery pseudoaneurysm, which was present before the cardiopulmonary resuscitation was done. Because the family opted for comfort care, further diagnostic methods were withheld.

Because these aneurysms can have fatal complications, definitive treatment options are necessary.^3^ The classic method is open surgical repair, but it is invasive and associated with complications of anesthesia-related risks, bleeding, wound infection, prolonged recovery, and death.^2^ On the other hand, targeted coil embolization is a minimally invasive operation that acts through 2 stages: embolization and surgical evacuation. Targeted coil embolization is an effective form of treatment for internal mammary artery pseudoaneurysms, especially those that are clinically minor.^1^ Another option is stenting the internal mammary artery via an endovascular approach.^16^

## CONCLUSION

This case describes a young patient who had a subdural hematoma and internal mammary artery pseudo-aneurysm, likely attributable to fibromuscular dysplasia. Although this patient's family decided on comfort measures, treatment methods are available for internal mammary artery pseudoaneurysms.

## Notes

*This article meets the Accreditation Council for Graduate Medical Education and the American Board of Medical Specialties Maintenance of Certification competencies for Patient Care and Medical Knowledge.*
